# Efficacy of AZM therapy in patients with gingival overgrowth induced by Cyclosporine A: a systematic review

**DOI:** 10.1186/1472-6831-8-34

**Published:** 2008-12-16

**Authors:** Marco Clementini, Gianluca Vittorini, Alessandro Crea, Maria Rosaria Gualano, Ludovica Antonella Macrì, Giorgio Deli, Giuseppe La Torre

**Affiliations:** 1 Periodontology Unit, Dental Institute, Faculty of Medicine, Catholic University of the Sacred Heart-Rome, Italy; 2 Epidemiology and Biostatistics Unit, Institute of Hygiene, Faculty of Medicine, Catholic University of the Sacred Heart-Rome, Italy

## Abstract

**Background:**

In daily clinical practice of a dental department it's common to find gingival overgrowth (GO) in periodontal patients under treatment with Cyclosporine A (CsA). The pathogenesis of GO and the mechanism of action of Azithromycin (AZM) are unclear. A systematic review was conducted in order to evaluate the efficacy of Azithromycin in patients with gingival overgrowth induced by assumption of Cyclosporine A.

**Methods:**

A bibliographic search was performed using the online databases MEDLINE, EMBASE and Cochrane Central of Register Controlled Trials (CENTRAL) in the time period between 1966 and September 2008.

**Results:**

The literature search retrieved 24 articles; only 5 were Randomised Controlled Trials (RCTs), published in English, fulfilled the inclusion criteria. A great heterogeneity between proposed treatments and outcomes was found, and this did not allow to conduct a quantitative meta-analysis. The systematic review revealed that a 5-day course of Azithromycin with Scaling and Root Planing reduces the degree of gingival overgrowth, while a 7-day course of metronidazole is only effective on concomitant bacterial over-infection.

**Conclusion:**

Few RCTs on the efficacy of systemic antibiotic therapy in case of GO were found in the literature review. A systemic antibiotic therapy without plaque and calculus removal is not able to reduce gingival overgrowth. The great heterogeneity of diagnostic data and outcomes is due to the lack of precise diagnostic methods and protocols about GO. Future studies need to improve both diagnostic methods and tools and adequate classification aimed to determine a correct prognosis and an appropriate therapy for gingival overgrowth.

## Background

Gingival overgrowth (GO) and gingival enlargement (GE) are terms used to indicate a pathologic increased volume of the gingiva instead of "hyperplasia" and "hypertrophy" [1], previously adopted.

Currently there are more than 20 pharmacological formulation that could be considered associated with GO [2-4]. These drugs can be divided into 3 categories: Calcium-channel blockers [5-8], anticonvulsants [9,10], immunosuppressants [11-16]. They all recognize gingival connective tissue as secondary target tissue. Reliable figures of prevalence of GO for these 3 different categories are not available in literature: table 1 shows the results of main epidemiological studies conducted in the USA.

**Table 1:** Estimated prevalence of Drug-Associated Gingival Enlargement according to the most frequently reported prevalence rates (Informational Paper: Drug-Associated Gingival Enlargement. *J periodontal 2004;75:1424–1431*)

**Category**	**Pharmacologic Agent**	**Trade Name**	**Prevalence**
**Anticonvulsants**	Phenytoin	Dilantin	50%
	
	Sodium valproate (valproic acid)	Depakene, Depacon, Epilim, Valpro	Rare
	
	Phenobarbitone	Phenobarbital, Donnatal	<5%
	
	Vigabatrin	Sabril	Rare
	
	Carbamazepine	Tegretol	None reported

**Immunosuppressants**	Cyclosporin	Neoral, Sandimmune	Adults 25–30% Children >70%

**Calcium channel blockers**	Nifedipine	Adalat, Nifecard, Procardia, Tenif	6–15%
	
	Isradipine	DynaCirc	None reported
	
	Felodipine	Agon, Felodur, Lexxel, Plendil	Rare
	
	Amlodipine	Lotrel, Norvasc	Rare
	
	Verapamil	Calan, Covera, Isoptin, Tarka, Verelan	<5%
	
	Diltiazem	Cardizem, Dilacor, Diltiamax, Tiazac	5–20%

Drug-induced GO has a multifactorial etiology [17]. The effects of the pharmacological molecules are modulated by these factors: plaque index [18] (directly correlated); gender (males are three times more sensitive); age (inversely correlated); drug's daily dose (directly correlated); HLAB37 positivity and simultaneous administration of corticosteroids or one of the 2 other categories [19]. All these factors act in various means synergistically enhancing GO [20]. The pathogenetic mechanism of drug-induced GO is still unclear. It's modulated by fibroblasts [21,22], cytokines [23-25], matrix metalloproteinases [26].

They seem to interact in a complex network to create the typical histopathological lesion of GO: an increased volume of the gingiva due to an abnormal stroma of connective tissue with overproduction of collagen and other extracellular matrix proteins, infiltrated by plasmacells and covered by parakeratinized epithelium also projecting irregular epithelial ridges deep inside [27].

In daily clinical practice of a dental department it's common to find GO in periodontal patients under treatment with Cyclosporine A (CsA), for example in transplanted subjects [28-32].

In our study a systematic review was conducted with the aim of evaluating the efficacy of Azithromycin (AZM) in patients with GO induced by assumption of Cyclosporine A (CsA).

The review question was:

- how effective is AZM in terms of clinical outcomes in patients with GO induced by assumption of CsA?

## Methods

In accordance with Needleman [33], a protocol was developed and it included all aspects of the review methods: inclusion criteria for studies, search strategy, screening methods, data abstraction, quality assessment by two independent reviewers and data synthesis.

### Inclusion criteria

#### Type of studies

Only Randomised Controlled Trials (RCTs) or systematic reviews were included because the RCT is the most appropriate design to choose for reviews of effectiveness of therapy.

#### Subjects

Only patients with a clinical diagnosis of GO induced by assumption of Cyclosporine A (CsA).

Type of treatment intervention: the intervention of interest is the use of Azithromycin (AZM) with and without scaling root planing (SRP); while the control groups received other systemic or local antimicrobials or placebo with and without SRP.

#### Types of outcome measures

Main change in clinical attachment level (CAL), mean change in probing pocket depths (PPD), changes in bleeding on probing (BOP), gingival index (GI) and plaque index (PI), changes in coronal tooth length or clinical crown length and in distance between interdental papilla and CEJ (cementum-enamel junction) were reported in this review as outcome measures.

### Search strategy

A research was performed using these online databases: MEDLINE (from 1966 to September 2008) Cochrane Oral Health Group Trials Register (September 2008); Embase (from 1988 to September 2008). The search strategy was as follows: our review question was divided in its main components to develop correct search terms for the databases, establishing a hierarchical order and using "text" terms, adopted by authors in the title and abstract, and code terms, adopted by the indexer of the database; the resulting key-words were: gingival overgrowth, Cyclosporine A, Azithromycin.

Then the search using key-words was performed into 2 different ways, using Boolean operators: "(gingival overgrowth OR GO OR gingival enlargement) AND (Cyclosporine OR Cyclosporine A OR CsA) AND (Azithromycin OR AZM OR AZT)" and without using Boolean operators: "gingival overgrowth CsA Azithromycin".

The search was limited to RCT studies conducted on humans and English language articles.

### Selection process

The initial selection process was realized independently by 2 researchers. The title, abstract, and key words of identified studies were screened independently by both reviewers for relevance.

No attempts were made at this time to identify studies that did not use adequate control group, because it was considered improbable that abstracts would report enough information about the setting. The subsequent selection was made to identify only articles that dealt specifically with the inclusion criteria.

### Quality assessment

The methodological quality for the included studies was assessed with a predetermined appraisal form, focusing the following issues: bibliographic details, details of the study setting as method of randomisation and blindness of patients, therapist and examiners, characteristics of study population, frequency and course of the interventions, baseline and outcome measures, completeness of follow-up.

Both reviewers independently assessed the quality of each study. The strengths and weaknesses of the study design of each included study were analysed. Disagreements on validity assessment were resolved by consensus and discussion.

## Results

The search identified 24 articles found in MEDLINE, 5 articles in EMBASE (already found in MEDLINE), 3 articles in Cochrane Central of Register Controlled Trials (already found in MEDLINE).

The critical analysis of these 24 papers showed that:

1. all studies are published in English language.

2. three articles don't answer to the review question because: in one the Authors try to outline a relationship between Chlamydia Pneumoniae infection and pathogenesis of increased gingival volume [34]; one study was conducted on animals [35]; in the third one the Authors suggest to treat CsA-induced gingival overgrowth administrating Tacrolimus, an alternative immunosuppressant, instead of Cyclosporine [36].

3. five are letters to editor or short communications about other previously published papers [37-41].

4. four are case reports [42-45].

5. one is a traditional review [46].

6. four are clinical trials [47-50].

7. two are controlled clinical trials (not randomised) [51,52].

8. five are randomised controlled trials (RCTs) [53-57].

In this systematic review were included only 5 RCTs excluding the remaining 19 studies.

### Studies Design

These five studies were RCTs and subjects were kidney transplant patients.

In the study published by Nash & Zaltzman [53], the efficacy of Azithromycin was examined in a double blind randomised crossover trial. 17 patients were randomised into 2 groups: one group received placebo for 5 days and after 2 weeks AZM (500 mg day 1, and 250 days 2–5) and the other group received AZM before placebo. Outcome measures were: gingival sulcus depths, tooth length and length of interdental papilla to the CEJ of two teeth in each of the four quadrants. The first group showed immediate improvement for the three analyzed periodontal parameters (pocket depth, tooth length, distance interdental papilla-CEJ) after receiving AZM, the second group had significant improvement immediately after AZM therapy but 3 weeks after there was a deterioration of the GD (gingival sulcus depth).

Besides the pharmacological therapy reduced the amount of gum bleeding and 67% of the patients reported that the treatment was at least somewhat useful.

In the study of Mesa et al. [54] 40 adult renal transplanted patients were included in a double blinded controlled randomised study. Patients were randomised into 3 groups (A: 13 subjects received MZN 250 mg 3 times for 7 days, B: 14 subjects received AZM 500 mg 2 times/die for 7 days, C: 13 received one cap of placebo 3 times daily for 7 days). Gingival and dental area were measured and the relationship between them was considered the GO index. The pretreatment GO index was 0.895 ± 0.16 for group A and after 30 days GO index was 0.897 ± 0.28, for group B was 0.932 ± 0.11 and after 30 days it became 0.909 ± 0.15, for group C was 1.073 ± 0.32 and at the end was 1.130 ± 0.30. After 30 days none of the patients with GO showed complete remission.

Morphometric index of GO, a particular kind of photographic analysis matching the gingival area and the tooth area, didn't show an improvement after the proposed pharmacological therapies; a 7-day course of Azytromicin or Metronidazole or placebo didn't produce statistical significative differences. Antibiotics seemed to act against bacterial over-infection and concomitant gingival inflammation.

In the study conducted by Nafar et al.[55] 25 patients were randomised into 4 groups (group 1 received systemic AZM on the first visit in the form of 6 caps, 2 on first day and 1 per day for 4 consecutive days, group 2 received placebo in the same way of group 1, group 3 received local AZM in form of oral gel for 1 week, group 4 received placebo in similar way to group 3).

Clinical parameters were bleeding on probing (BOP), GO index and plaque length (PL). Biometric parameters were probing pocket depth (PPD), crown length (CL) and stent interdental papilla (IDP).

Only BOP index showed an improvement after 2 or 6 weeks of the 4 therapies performed. But during the first visit all patients received a professional scaling and root planing using ultrasonic cavitron after the administration of 2 gr. of Amoxicillin: in this way local periodontal irritative deposits were removed. The other adopted periodontal parameters didn't show significant differences.

In the study published by Chand et al. [56] 25 patients (mean age of 17.48 years) were included in a double blinded randomised study and divided into 2 groups: the first one received AZM 500 mg on the first day and 250 mg days 2–5, the other group received Metronidazole (MNZ) 45 mg/kg/die divided into 3 doses daily for 7 days. Baseline gingival measurements were 12.87 ± 0.40 mm for AZM group and 13.13 ± 0.29 mm for MNZ group. After 24 weeks gingival measurements were 11.50 ± 0.14 mm for the first group and 12.23 ± 0.12 mm for the second. The most significative difference was found at 4 weeks: at this time Azytromicin group was significantly more effective than Metronidazole.

In the study published by Ramalho et al. [57] 20 renal transplanted patients with GO were enrolled and randomised into two groups: group A received AZM 500 mg for 3 days with oral hygiene program for 30 days, while the control group received only the oral hygiene program. Outcome measures were: subjective evaluation of oral hygiene, Simplified Oral Hygiene Index (SOHI) [58] and GH index [59].

About the first outcome measure patients in both groups declared to have improved their dental condition, in fact they had less pain and bleeding. For the other outcome measures, after 15, 30 and 60 days patients in AZM group showed statistically significant improvement of SOHI and GH index.

In the data summary table 2 patients enrolled, therapy administered, study location, study design, duration, outcomes measures and results are available.

**Table 2:** Studies characteristics

**Study**	**Chand DH et al. 2004**	**Nafar M et al. 2003**	**Mesa FL et al. 2003**	**Nash et al. 1998**	**Ramalho et al. 2007**
**Participants**	25 (17 m, 8 f) mean age: 17.5 ± 6.06 ys	25 (16 m, 9 f) mean age: 40 ys (range 15–60)	40 (? m, ? f) mean age: 41.4 ys (range 24–60)	17 (12 m, 5 f) mean age : none specified	20 (f) mean age: 32 ± 3 ys

**Setting**	Cleveland Clinic Found., Cincinnati Children's Medical Centre	2 medical centres in Teheran	University hospital of Granada	None specified	University Ospital of Sao Jose do Rio Preto

**Interventions**	**A**: AZM 500 mg day 1, 250 mg. days 2–5 **B**: MNZ 45 mg/kg/die, 3 doses daily for 7 days.	(All, during the first visit: SRP + 2 g amoxicillin) **A**: AZM 500 mg day 1, 250 mg days 2–5 **B**: placebo for 5 days. **C**:AZM gel 25% for 7 days. **D**: placebo for 7 days.	**A**: MNZ 250 mg 3 times/die for 7 days. **B**: AZM 500 mg 2 times/die for 7 days. **C**: placebo 3 times/die for 7 days.	**A**: placebo for 5 days + (after 2 weeks) AZM 500 mg day 1, 250 mg days 2–5 **B**: AZM 500 mg day 1, 250 mg days 2–5 + (after 2 weeks) placebo for 5 days	**A**: oral hygiene program **B**: oral hygiene program + AZM 500 mg for 3 days

**Study Design**	Double Blind RCT	Double Blind RCT	Double Blind RCT	Double Blind RCT	RCT

**Length**	24 weeks	6 weeks	4 weeks	12 weeks	30–60 days

**Outcomes measures**	Pocket Depth (mean), photographs	Bleeding on Probing, GO index, Plaque Length, Pocket Depth, crown length, stent interdental papilla	Morphometric GO index, serum creatinine and creatinine clearance	Pocket Depth, tooth length, length of the interdental papillae to the cementum enamel junction, questionnaire, photographs	Subjective evaluation of oral hygiene (questionnaire), SOHI, GH index

**Results**	*Baseline GO:* **A**: 12.87 +/- 0.40 mm **B**:13.13 +/- 0.20 mm *After 24 weeks:* **A**: 11.50 +/- 0.14 mm **B**: 12.23 +/- 0.12 mm	Significantly results only for BOP. Insignificant differences between drug and placebo groups for other parameters	Not statistically significant results	Significant improvement in all 3 types of periodontal measurements after assumption of AZM	Significant improvement of SOHI and GH index in patients in AZM group

## Discussion

Analysing the eligible studies it arose heterogeneity in various aspects of studies design such as patients characteristics (mean age for example), timing of therapy and follow up, kind of therapy put in comparison (AZM with MZM or Placebo) and drug's daily dose. Also a relevant heterogeneity of diagnostic tools and outcome measures was highlighted: in every study different parameters such as pocket depth, bleeding on probing, plaque index, photographic analysis, interdental papilla height, tooth length were used to evaluate the degree of GO. Evaluating the results, the difficulty in pooling data from these selected studies was evident: consequently it was impossible to perform a meta-analysis with sensitivity analyses because the multiple differences between them and their insufficient number impaired a statistical combination of data. The only way to make sense of the different results from the selected studies was the construction of a data summary table.

Anyway, AZM, only if associated with SRP, could be considered a better way to treat GO caused by CsA therapy than MZM or placebo such as it arose considering these 5 eligible studies. In fact in the studies of Nash et al., Nafar et al. and Chand et al. subjects treated with AZM associated with calculus and plaque removal had improvement of their condition of GO with statistically significant results. In the study of Ramalho et al. AZM associated with oral hygiene program reduced GO. Only in Mesa et al. study there were not significant differences using AZM instead of MZM or placebo.

One of the most recent papers on this topic [52] (Argani et al. 2006) was considered very interesting but it was not eligible because it was lacking of randomisation and blindness. In fact the Authors examined the efficacy of local Azithromycin, in the form of toothpaste, against cyclosporine-induced gingival overgrowth, reducing the patients compliance and the adverse effects of a systemic therapy.

A well-designed randomised multicentric clinical trial would be recommendable to enlarge the sample so that results could be generalized.

Anyway, the pathogenesis of GO as well as the mechanism of action of AZM still remains unclear. The great heterogeneity of diagnostic data and results in our systematic review is due to the lack in literature of complete RCTs on the efficacy of systemic antibiotic therapy of GO, mainly due to the absence of precise diagnostic methods and tools and of an adequate classification aimed to determine a correct prognosis and an appropriate therapy for GO.

### Future projections

Future researches are needed to outline a correct classification and to use universally accepted, unambiguous diagnostic parameters and an ideal study design like a RCT: in this way it is possible to identify an efficacious GO management.

## Conclusion

In literature there are few RCTs on the efficacy of systemic antibiotic therapy of GO and it is mainly due to the absence of precise diagnostic tools and of an adequate classification aimed to determine a correct prognosis and an appropriate therapy for GO. The pathogenesis of GO as well as the mechanism of action of AZM still remains unclear: future researches are needed.

## Competing interests

The authors declare that they have no competing interests.

## Authors' contributions

MC, GV, AC, LAM carried out bibliographic search and selected the articles; GD carried out the coordination; GLT and MRG participated in the design of the study and methodology.

All authors read and approved the final manuscript.

## Pre-publication history

The pre-publication history for this paper can be accessed here:


